# Allograft Labral Reconstruction of the Hip: Expanding Evidence Supporting Greater Utilization in Hip Arthroscopy

**DOI:** 10.1007/s12178-022-09741-y

**Published:** 2022-02-10

**Authors:** Brian J. White, Shannon M. Constantinides

**Affiliations:** 1 Western Orthopaedics, Denver, CO USA; 2 Porter Adventist Hospital, Denver, CO USA; 3 Colorado Center of Orthopaedic Excellence, Colorado Springs, CO USA; 4 Rock Harbor Research Institute, 13 Corrine Place, Key Largo, FL USA

**Keywords:** Labral reconstruction of the hip, Allograft labral reconstruction

## Abstract

**Purpose of Review:**

The current review investigates outcomes and failure rates associated with arthroscopic circumferential allograft labral reconstruction of the hip, both as a revision and primary procedure in treating femoroacetabular hip impingement and labral-related pathology.

**Recent Findings:**

Numerous studies within the last decade have demonstrated excellent patient-reported outcomes, high rates of return-to-play in athletes, and low failure rates in patients having undergone arthroscopic circumferential allograft labral reconstruction of the hip. Removal of chronically diseased and injured labral tissue can eliminate a significant pain-generator from the hip joint. Additionally, circumferential reconstruction of the labrum restores the hoop fiber strength and fluid seal akin to what would be seen with native, healthy labral tissue. Recent research has shown that arthroscopic circumferential allograft labral reconstruction may be used not only in the revision setting, but as a primary procedure. Circumferential labral reconstruction should be considered when a surgeon feels that the labrum is irreparable or has failed previous repair.

**Summary:**

Arthroscopic circumferential allograft labral reconstruction of the hip can be utilized as treatment option not only in revision settings, but also in primary treatment for femoroacetabular impingement and labral pathology

## Introduction

While initially designed to resect damaged soft tissue, the goal of modern hip arthroscopy has shifted focus to preserving and restoring function within the hip joint and improving its biomechanics. Labral repair, which has been considered the standard treatment for hip impingement and labral tears, is becoming much more commonplace. However, considering a growing body of evidence demonstrating positive outcomes and low revision rates, we contend that labral reconstruction can be considered a primary treatment for femoroacetabular hip impingement (FAI) and labral tears, especially in such circumstances in which the labral tissue is deemed irreparable. A number of factors can contribute to poor healing with labral repair, thus, decreasing the labrum’s ability to provide a functional fluid seal around the joint, and increasing the likelihood of continued pain, intra-articular injury, and joint dysfunction [1]. As such, labral reconstruction offers clear advantages including removal of unhealthy, painful labral tissue; complete access for reshaping the acetabulum and addressing pincer impingement; and incorporating a graft that will mimic the biomechanical benefits of a healthy, native labrum^1^. This article will provide a brief overview of our progress as a specialty, evidence to support current practices as they pertain to arthroscopic circumferential allograft labral reconstruction of the hip, and implications for future directions in hip preservation surgery.

## Historical Perspectives

Due to an exponential surge in demand, the last two decades have seen unprecedented growth within the specialty of arthroscopic hip surgery. Used as a technique to treat intra-articular pathology, including FAI and labral tears, hip arthroscopy has become increasingly common. Data from 2006 to 2010 demonstrated a 600% increase in the number of arthroscopic hip surgeries being performed annually in the USA [2,3], with a study by Bonazza and colleagues, which queried a large national data base, reporting that from 2008 to 2013 the number of arthroscopic hip procedures per patient increased by 378%. Considering that labral reconstruction is somewhat still in its adolescence, more research is needed to calculate quantifiable trends in the occurrence of this procedure. However, the last few years have shown a noticeable increase in the numbers of studies focusing on labral reconstruction due to technological and evidence-based innovations in surgical techniques and largely promising and positive outcomes data [4–16]. Six meta-analysis or systematic reviews have been published in the last few years, alone, and point to equivalent or improved results with labral reconstruction compared to repair (Table 1) [7–9,11,17,18]. Likewise, at least 17 studies have been published within the last decade evaluating various outcomes of arthroscopic labral reconstruction using allograft [4,5,9,10,12–14,16,19–27] and autograft [28–36] techniques (Table 2). Overall, current evidence has concluded that arthroscopic labral reconstruction of the hip is associated with improved pain and functional status, low rates of complications or need for revision surgery, and rare progression of arthritis.

**Table 1:** Systematic reviews/ meta-analyses on labral reconstruction 2019–2021

	***N*** **(studies)**	***N*** **(hips/patients)**	**Graft**	**M age** **(years)**	**M follow-up** **(months)**	**Convert to THA**	**M improvement in mHHS**
Al Mana et al. 2019^17^	9	265 hips	Allo (5)	35	37 (12–61)	5.70%	28 (mHHS)
			Auto (4)				
Bessa et al. 2020^18^	7	402 patients	Auto	44 (16–72)	66 (12–120)	0–13%	28 (mHHS)
Maldonado et al. 2020^9^	10	582 hips	Allo	30 (27–52)	45 (24–66)		39 (mHHS)
Rahl et al. 2020^7^	8	537 hips	Auto	37	29	0–13.2% (Auto)	29 (mHHS)
						0–12.9% (Allo)	
Safran et al. 2021^11^	7	228 hips	Not	38	35	3%	PROs reported as improved;
			Reported				numeric data not reported
Trivedi et al. 2019^8^	11	373 patients	Allo (4)	36.6 (28–43)	12	0–23%	24 (mHHS)
			Auto (6)				
			Mixed (1)				

*Auto* autograft, *Allo* allograft, *THA* total hip arthroplasty, *PRO* patient-reported outcome, *mHHS* modified Hip Harris score

**Table 2:** Published *arthroscopic* labral reconstruction outcomes

**Study**	**CR v. SR**	**Graft**	***N***	**Sex**	**M age, years**	**M follow-up, months**	**Convert to THA**	**M improvement in mHHS**
**Allograft**								
Bodendorfer et al. 2021^19^	CR and SR	ITB, Hamstring	51 CR	187 m	42.3 (31–54)	25	2 (4%)	17 CR
			52 CR	229 f				22 SR
Carreira et al. 2018^20^	SR	TFL	31	11 m	44 (20–66)	12	4 (13%)	21
				20 f				
Chandrasekaran et al. 2017^21^	SR	Hmastr. Auto	22	8m	32 (22–42)	12	1 (4%)	11
		Hamstr. Allo		14 f				
Chen et al. 2021^46^	CR	MM	7	1 m	47 (22–57)	12	None Reported	Not reported:
				6 f				Improved ROM/MAPs
Domb et al. 2021^12^	CR	Frozen Allo.	26	10 m	25 (22–44)	24	1 (3%)	17
				16 f				
Domb et al. 2020^13^	CR	AT	37	18 m	47 (42–48)	24	2 (5%)	24
				19 f				
Domb et al. 2019^22^	SR	Not Reported	28	12 m	35 (15–69)	60	Reported graphically	20
				11 f				
Maldonado et al. 2019^23^	Not Reported	Hamstr. Auto	13 Auto	16 m	36 (18–56)	24	1 Auto (7%)	16 Auto
		Hamstri. Allo	28 Allo	13 f			3 Allo (10%)	19 Allo
Maldonado et al. 2021^14^	CR, SR	Semi-T	47 CR	62 m	45 (34–55)	28	3 CR (6%)	22 CR
			80 SR	65 f	43 (33–55)		5 SR (6%)	20 SR
Maldonado et al. 2020^10^	Not reported	Hamstring	32	18 m	40 (16–59)	28	Not Reported	22: 75% return to sport
				14 f				
Rathi and Mazek, 2017^24^	SR	TFL	10	10 m	35 (26–44)	23	None Reported	37
				0 f				
Scanaliato et al. 2020^16^	CR	TFL	30	13 m	30 (16–56)	24	None Reported	Not reported:
				17 f				86.7% returned to play
Scanaliato et al. 2018^25^	CR	TFL	63	23 m	43	24	2 (3%)	21
				40 f				
White et al. 2020^4^	CR	ITB	270	53 m	41 (30–65)	44	10 (4%)	37
				27 f				
White et al. 2018^26^	CR	ITB	58	6 m	33 (15–52)	56	None reported	30
				23 f				
White et al. 2016^5^	CR	ITB	90	26 m	35 (16–60)	28	Not reported	32
				72 f				
White et al. 2016^27^	CR	ITB	152	64 m	39 (16–58)	28	13 (10%)	34
				78 f				
**Autograft**								
Amar et al. 2018^28^	CR	RF	22	19 m	42 (22–68)	32	2 (9%)	25
				12 f				
Boykin et al. 2013^29^	SR	ITB	21	19 m	28 (19–41)	41	2 (10%)	17
				0 f				
Domb et al. 2014^30^	SR	Gracilis	11	7 m	33 (18–45)	26	None reported	27
				4 f				
Geyer et al. 2013^31^	SR	ITB	76	42 m	39 (18–64)	49	19 (25%)	24
				33 f				
Lebus et al. 2018^32^	SR	ITB	317	170 m	35 (15–71)	44	41 (13%)	20
				141 f				
Matsuda and Burchette, 2013^33^	SR	Gracilis	8	7 m	35 (18–58)	30	Not reported	Not reported:
				1 f				Improved NAHS
Philippon et al. 2020^34^	SR	ITB	187	110 m	35	44	27 (14%)	Pre-op not reported
				77 f				
Philippon et al. 2010^35^	SR	ITB	47	32 m	37 (18–55)	18	4 (9%)	23
				15 f				
Rathi and Mazek, 2018^36^	SR	RF	7	5 m	35 (25–41)	15	None reported	37
				2 f				

*m* male, *f* female, *mHHS* modified Hip Harris score, *NAHS* non-arthritic hip score, *CR* circumferential reconstruction, *SR* segmental reconstruction, *ITB* iliotibial band, *TLF* tensor fascia lata, *MM* medial meniscus, *RF* rectus femorus

## Labral Function

Within the last decade, a number of cadaveric studies have demonstrated the importance of the acetabular labrum in preserving normal hip function [37–41]. Composed of a complex fibrocartilaginous matrix, the hip labrum in combination with the transverse acetabular ligament form an uninterrupted ring around the acetabulum[40]. During weight bearing and with hip range of motion, this tissue ring is exposed to forces exerting compression and elasticity in axial, load-bearing and circumferential directions [40]. The factors that allow the labrum and transverse ligament to endure such forces are what allow it to increase weightbearing surface area and evenly distribute contact pressure off the cartilage of the femoral head and acetabulum^40^. Furthermore, the labrum functions to preserve intra-articular fluid pressure within the femoroacetabular joint [40]. By creating a fluid seal, an intact labrum helps maintain the fluid pressurization required for stability of the hip against distraction forces and protection of the intra-articular cartilage matrix [37–40]. Labral tears as well as an insufficient labrum have been shown to be associated with loss of fluid pressurization within the joint [37–39]. Biomechanical research regarding these pathophysiological issues has demonstrated that labral reconstruction can restore the fluid seal, thus, restoring fluid pressurization and stability within the joint, and decreasing the damaging contact pressure and friction on the articular surfaces [37–39].

## Indications for Hip Labral Reconstruction

While initially seen as a salvage procedure, our recommendation for primary labral reconstruction reflects the culmination of growing evidence supporting its benefit in revision settings and as a primary procedure to restore and preserve function when the labrum is deemed irreparable. This may include circumstances when labral tissue is compromised or otherwise inadequate or inappropriate for a repair, such as would be seen with congenitally or acquired labral deficiency, hypertrophic labra, ossified labra, or labral tissue that has been damaged, scarred, or compromised from previous surgery. In a 2020 cadaveric study, for example, Storaci and colleagues [40] found that when compared to larger labral (> 6mm), smaller labra were associated with a higher risk of suction seal rupture within the femoroacetabular joint.

In a 2019 systematic review [17], Al Mana and colleagues found that the most commonly cited indications for labral reconstruction included non-functional, ossified, or irreparable labra in young people with little or no chondral wear. The data presented was supported by findings from a recent study presented by Mayo et al. [42], wherein machine learning technology, or artificial intelligence-based algorithmic data science, was used to detect indications for arthroscopic labral reconstruction. The most frequently cited indication was severe labral damage and the presence of calcified labral tissue. Additionally, a 2018 survey of 12 hip arthroscopy specialists, in accordance with other recently published literature, cited ossified labral tissue, poor-quality labral tissue, insufficient labral tissue, and irreparable labral tissue as the most common indications for choosing labral reconstruction over repair [8,9,17,23,25,26].

## Arthroscopic Technique: Graft Choice and Circumferential Reconstruction

### Graft Choice

The technique for labral reconstruction which we described in 2016 [6] has been slightly modified to include use of a longer graft, thus, ensuring an uncompromised and truly circumferential seal around the femoral head in all four quadrants of the acetabulum. When discussing labral reconstruction, we do so in reference to circumferential, total, or complete labral reconstruction, as opposed to segmental labral reconstruction. While segmental labral reconstruction is technically less challenging to perform, it has disadvantages.

First and foremost, the fundamental flaw with segmental labral reconstruction is that a shorter, roughly 4-cm graft is often placed in the anterosuperior quadrant which is the highest stress zone of the acetabulum. In this location and because the graft is short, it does not have surrounding attachments posteroinferiorly and anteroinferiorly. As it lacks surrounding support, this location of the segmental reconstruction can make healing and incorporation of the graft more challenging. This disruption of the circular structure of the labrum results in decreased hoop fiber strength of the remaining labral remnant. Unfortunately, there is no way to affix the segmental graft to the native labrum as the fibers run in parallel directions and suturing between the two structures often does not hold. Longer, circumferential grafts negate these issues as they span the distance from the origin of the anterior transverse acetabular ligament to the postero-interior acetabulum and cover all four quadrants of the acetabulum. Much like a suspension bridge, circumferential grafts are stronger. They provide rigid fixation antero-inferiorly and posteroinferiorly to give greater support to the critical, high-stress antero-superior acetabulum. In contrast, segmental grafts are placed in this zone of the acetabulum without surrounding support. The circumferential graft can reproduce the native fluid seal around the femoral head and thereby can more evenly distribute the forces associated with weight bearing and range of motion. Second, by removing only a segmental section of the labrum, the remaining, unhealthy, and highly innervated native posteroinferior and anteroinferior labral remnants remain in the joint and are vulnerable to further tearing and pain generation.

In addition to recommending circumferential labral reconstruction, we recommend the use of a frozen fascial allograft (AlloSource) [15]. Our preference was also substantiated by other surgeons in a recently published review, where 91.7% of high-volume hip arthroscopists reported a preference for use of allograft over autograft when performing labral reconstruction^23^. Likewise, in reviewing literature on labral reconstruction over the past decade, we found that the majority of study protocols, especially within the last few years, utilized allograft tissue (Table 2). Surgeon preferences for allograft included hamstring, fascia lata, anterior tibialis, and tissue bank acetabular labrum [23]. Other recent literature has documented use of the peroneus brevis [43–45] and medial meniscus [46] as alternate sources of allograft tissue. In a 2020 systematic review and meta-analysis, Rahl et al. [7] found that in regard to allograft choice, the most commonly utilized tissues included iliotibial band (76.2%) and tensor fascia lata (23.8%). It is important to note that several studies on labral reconstruction transitioned mid-study in protocol from autograft to allograft due to issues related to donor site morbidity. In a 2019 study investigating differences between auto and allograft in labral reconstruction, Maldonado and colleagues found slightly higher post-op modified Hip Harris scores (mHHS), and an improved mean mHHS (19 points) in their allograft group when compared to their autograft group [47]. In the conclusion of their study, Maldonado et al. stated that donor site morbidity in the autograft cohort may contribute to higher patient satisfaction in the allograft group. This was also consistent with our early experience, as donor site morbidity of the iliotibial band harvest site became the main driver in the direction of pursuing allograft.

### Circumferential Reconstruction

**Fig. 1:**
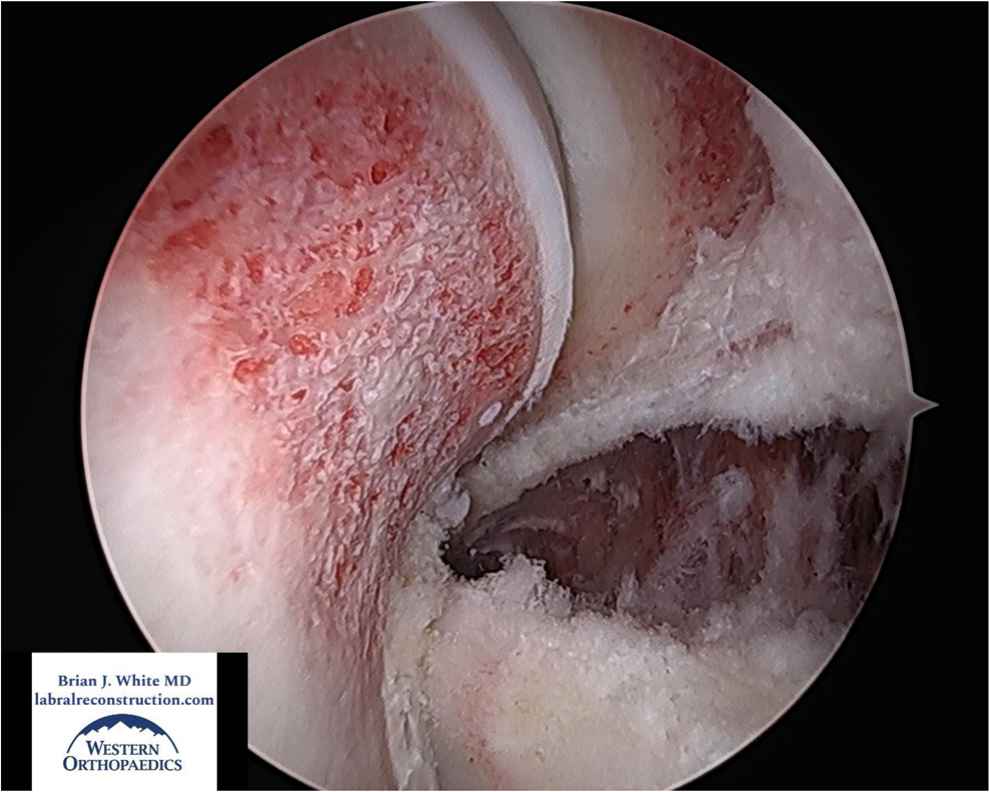
View from the anteromedial portal in a left hip of a proper, complete femoral osteoplasty with an anatomic shape and an alpha angle in the mid 40s. Also seen is the pincer resection as the joint is reduced

**Fig. 2:**
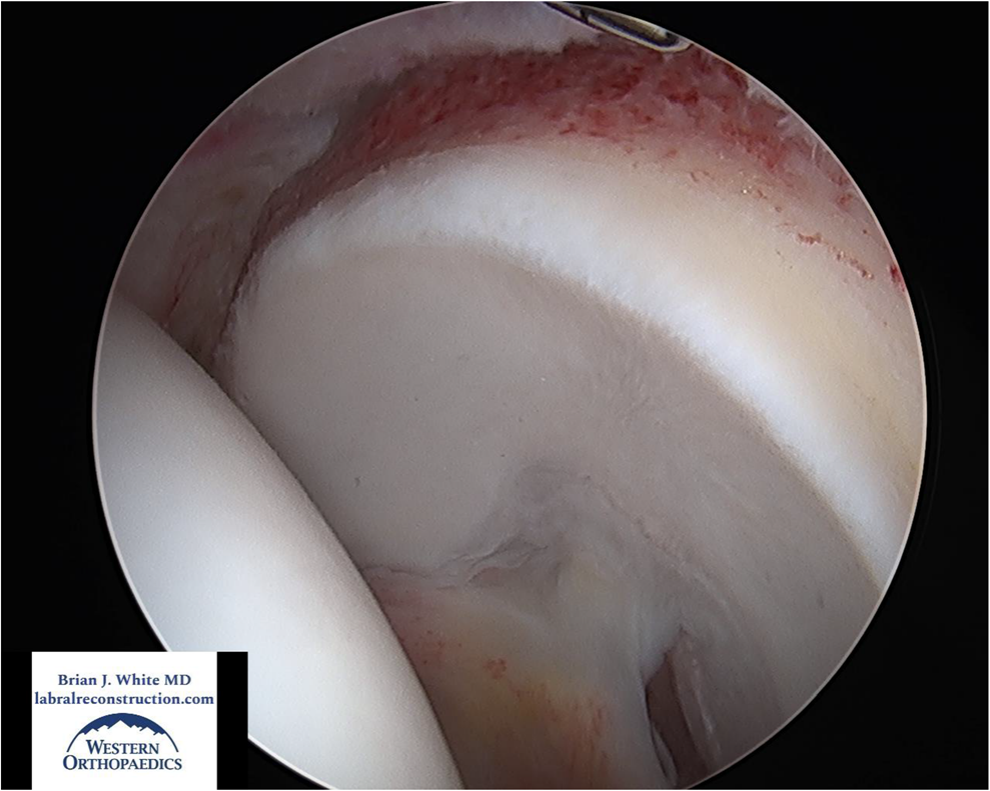
View from the anterolateral portal in a left hip of a well-prepared acetabular rim. The pincer was resected, and the center edge angle was improved from 42 degrees to 34. The cartilage was also well stabilized

Perhaps the most critical aspect of the procedure involves the meticulous treatment of the boney morphology causing the femoroacetabular impingement (FAI). During this stage of the operation, the femoral head neck junction (cam morphology) is reshaped to an anatomic, natural shape that will fit properly into the acetabulum. This eliminates impingement of the femoral head neck junction against the labral graft and improves both flexion and rotation of the hip. The acetabulum is circumferentially excoriated to prepare for biologic incorporation of the labral graft and pincer morphology, or over coverage, is removed when appropriate (Figures 1 and 2). If a pincer lesion is truly absent or in the case of acetabular dysplasia, the acetabular rim must be carefully prepared with a burr to create a bleeding response that will perpetuate osseous integration of the graft into the acetabular rim. It is critically important in this type of hip that great care be taken to excoriate the edge of the acetabulum without reducing the acetabular volume. In general, we recommend that 1mm or less of bone be resected in the presence of a normal or low volume acetabulum to prepare for biologic incorporation of the labral graft. The cartilage on the acetabular edge is also stabilized and beveled to protect it from further injury and delamination. Following the conclusion of the bony work and preparation, torn or degenerative labral tissue is removed from its origin at the transverse acetabular ligament to the posteroinferior acetabulum. For purposes of orientation, the area from which labral tissue is resected spans an anteroinferior position (7:30 left hips and 4:30 right hips) to a posteroinferior position (4:00 left hips and 8:30 right hips).

**Fig. 3:**
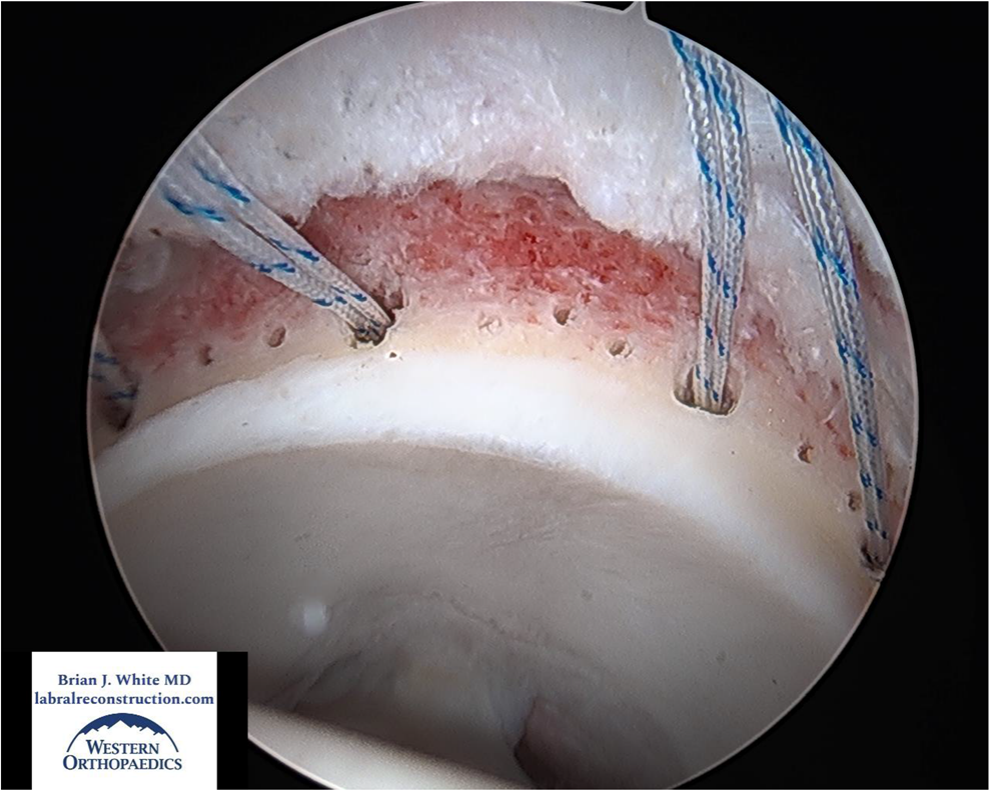
View from the anteromedial portal in a left hip of “vascular channels” 0.6 mm in diameter between anchors to encourage healing and incorporation of the graft

With both sides of the joint reshaped and prepared, suture anchors are placed measuring 10 to 12 mm apart around the entire acetabular rim (Q-Fix, Smith & Nephew). Anchors are placed from one of two direct anterolateral (DALA) portals, which allow access to both the anterior and posterior acetabulum. To avoid eversion of the labral graft, suture anchors should be placed as close to the cartilage border as possible. It is helpful to place all suture anchors before the acetabular graft is introduced into the joint to allow for optimal visualization and anchor positioning. After all suture anchors have been placed, and before the graft is introduced into the joint, two small drill holes measuring approximately 0.6 mm in diameter are placed between each anchor site. The drill holes create vascular channels which foster osseous integration of the graft into the acetabular rim and are particularly helpful in areas where only the acetabular edge could be excoriated to avoid loss of cup volume (Figure 3).

Graft length is determined by measuring the length of the labral defect from front-to-back with a 4-mm-wide elevator. Approximately 3 to 4 cm are then added to the measurement to account for the nonlinear contour of the acetabular rim. After graft length has been calculated, the graft is meticulously prepared by tubularizing the fascial tissue. This is accomplished by folding the fascia into thirds or quarters and then passing a 2-0 Vicryl suture through several small bites in an accordion-like manner at the end of the graft. These are tied, and the suture tails are then attached to a graft master to maintain adequate tension on the graft. Using a 2-0 Vicryl suture, a baseball stitch is run from front to back through the graft, using intermittent circumferential wraps, to compress the tissue.

The preferred diameter of the final graft varies among surgeons. A larger diameter graft is easier to achieve a seal with the femoral head but takes up space during the operation and is harder to compress and achieve incorporation. In contrast, a smaller diameter graft is easier to compress and incorporate, but it is harder to obtain a final seal between the graft and the femoral head. Over the years, we have found that a final graft measuring 5 to 5.5 mm in diameter provides adequate material to establish a seal with the femoral head and can be rigidly fixed and compressed. This size also appears aesthetically proportionally appropriate.

Using a cannula, the graft is brought into the joint via the anterior DALA portal. A suture limb from the most antero-inferior anchor is tied directly to the graft in figure of eight fashion. With the graft fixed to the suture, the anchor is used as a pulley to advance the graft into the joint. Once in the joint, the graft is placed in provisional position around the acetabular rim with a probe. Once positioned, a probe through the anteromedial (AM) portal allows the surgeon to maintain tension on the graft. Sutures are secured for the first two to three anchors. The most difficult area to create a seal between the graft and the femoral head is the antero-superior zone as it represents a challenging transition for the rigid graft from the vertical anterior wall of the acetabulum to the horizontal/lateral zone of the acetabulum. To offset the potential of not having a seal between the graft and the femoral head in this section, the suture anchors are passed, but not tied, at the anterior-superior, lateral, and posterior positions around the acetabulum. The graft is then fixed posteroinferiorly to tension the graft so that it can follow the curvature of the antero-superior acetabulum.

**Fig. 4:**
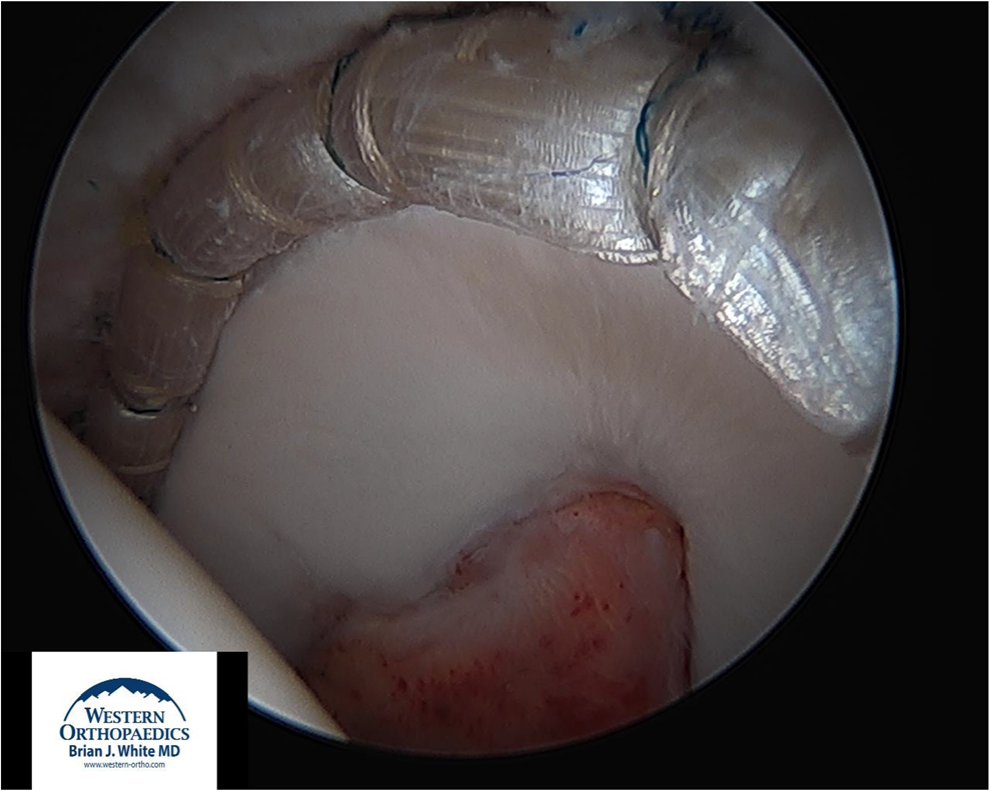
View from the anterolateral portal of a left hip showing the anterior portion of a 13-cm allograft labral reconstruction fixed with 13 anchors

**Fig. 5:**
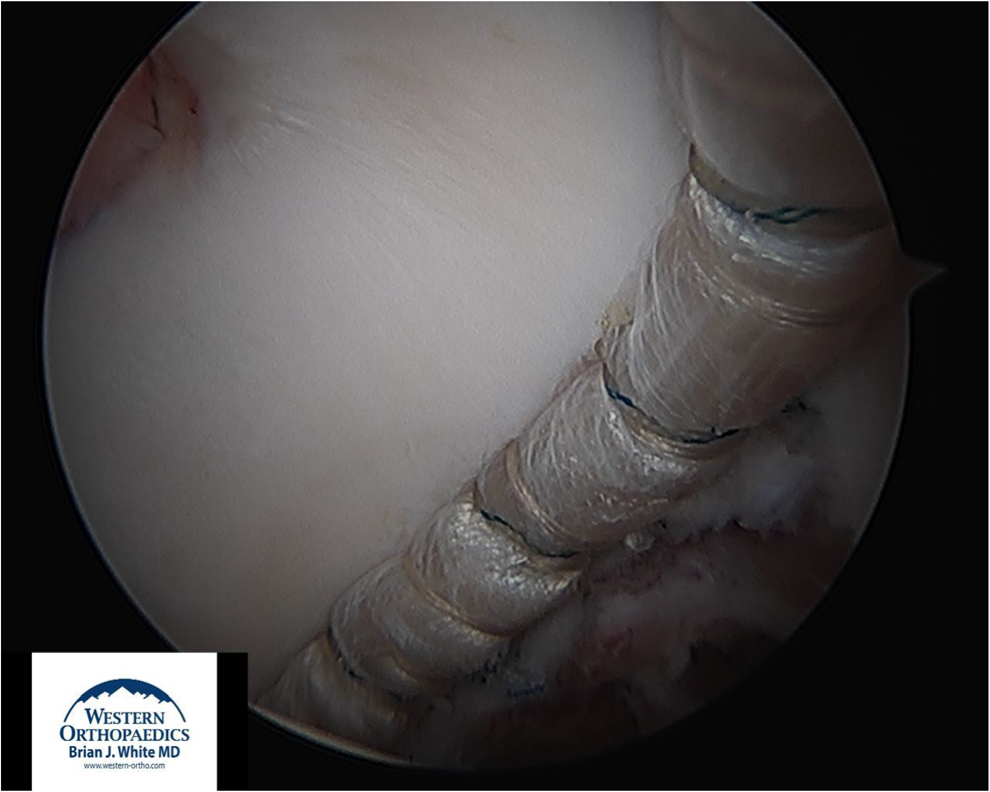
View from the anteromedial portal of a left hip showing the posterior portion of the same 13-cm labral reconstruction

After all sutures have been passed, the graft is tensioned and cut posteroinferiorly. It is important to note that the graft is cut in the joint to ensure that its length is appropriate. This is the advantage of the Front-to-Back technique as graft length is tailored in situ to avoid a mismatch between the graft length and the length and contour of the acetabulum [6]. For longer grafts, an additional portal (posterior and proximal to the antero-lateral [AL] portal) may be required to cut the graft. Using a grasper inserted through the AL portal to hold tension on the graft, the graft is cut using a beaver blade. At present, the length of a reconstructed labral allograft measures between 11.5 and 14.5 cm in length (Figures 4 and 5).

**Fig. 6:**
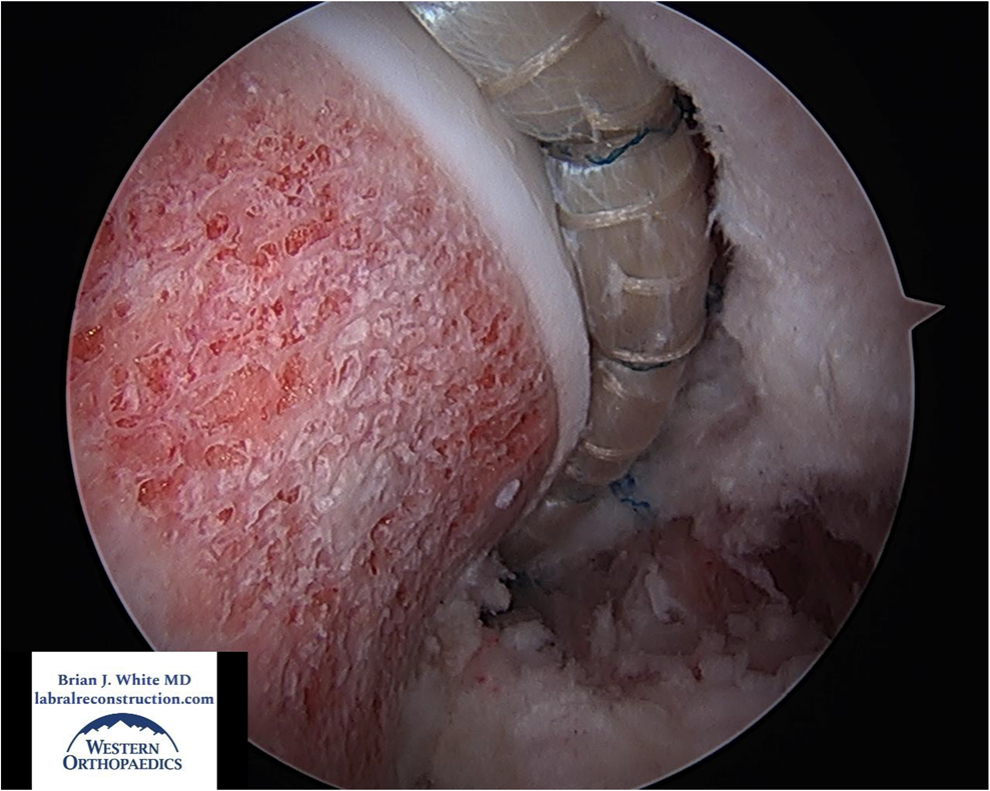
View from the anteromedial portal of a left hip with the joint reduced and an anatomic seal formed between a 12.5-cm allograft labral reconstruction and the femoral head

At the most posteroinferior aspect of the acetabulum, two anchors are placed only a few millimeters apart. Using an Elite Pass (Smith & Nephew), the sutures from the distal anchor are passed through the graft and are then tied, while the sutures from the adjacent anchor are tied circumferentially around the graft. With the graft now fixed antero-inferiorly and posteroinferiorly, the sutures in between can be secured. This is done in the peripheral compartment with the hip joint reduced or off traction. The camera is then moved to the AL portal and a cannula is positioned in the posterior DALA portal. The hip is then taken off traction which reduces the graft to the rim of the acetabulum and relieves tension on the anchors. Remaining sutures are tied in the peripheral compartment to ensure rigid fixation of the graft. It is absolutely critical that the graft form a perfect seal around the femoral head (Figure 6). Once the graft is rigidly fixed, dynamic testing under direct arthroscopic visualization is performed with the hip flexed and internally rotated. This is done to confirm that there is no graft impingement or joint instability. The hip capsule is closed using a #1 Vicryl or permanent suture. The extent of capsular closure, which may include one versus two sutures, is determined by the baseline degree of capsular laxity.

## Outcomes

Publications reporting on the outcomes of arthroscopic allograft labral reconstruction have continued to increase, with eight new studies having been published in 2020–2021, alone, on the topic [4,10,12–14,16,19,46]. Overall, evidence has shown highly positive patient-reported outcomes, low failure rates, and significant improvements in functional return to sports (Table 2). In the studies we reviewed, circumferential reconstruction was the predominantly utilized technique, and allograft preferences included iliotibial band, anterior tibialis, hamstring, and tensor fascia lata. Where conversion to total hip arthroplasty (THA) was a measurable outcome, only 142 of 1823 hips (7%) were reported to have converted within the 1 to 2 years following surgery [4–6,12–14,19,20,24–26,46]. Among the hips that did not fail, authors reported improvements on a wide variety of validated patient-reported outcome (PRO) measures, range of motion, and radiographic correction of bony hip impingement morphology [4,10,12–14,19,25,46]. Where assessed, the mHHS showed an average improvement of 24 points [4–6,10,12–14,19,20,25,26,36]. Likewise, in a 2020 study on labral reconstruction in competitive athletes, Scanaliato [16] and colleagues found that all athletes reported substantial clinical benefit, with 87% of athletes returning to play in an average of 6.6 months. These findings were similar to Maldonado et al. [10] who found that 78% of athletes returned to sport following primary arthroscopic labral reconstruction. Overall, evidence points to both primary and revision hip arthroscopy with labral reconstruction to be a highly successful operation.

### Revision: Reconstruction Versus Revision Repair

Those who have failed a previous hip arthroscopy represent a challenging patient population and there has not been an evidence-based consensus on the best approach for them. As such, in 2016 we compared outcomes between patients who underwent revision hip arthroscopy with iliotibial band allograft labral reconstruction versus labral re-repair [5]. In our retrospective “repair or reconstruct” cohort study, we followed 113 hips (*n* = 15 re-repair, *n* = 98 reconstruction) which had previously undergone previous labral repair or debridement^5^ for an average of 2.4 (reconstruction) to 4.7 (revision repair) years. Hips that underwent revision arthroscopy with labral revision-repair were 4.1 times (*n* = 7, 50%; 95% CI 1.9–8.8; *p* < .01) more likely to fail treatment when compared to patients who underwent revision arthroscopy with labral reconstruction (*n* = 11, 13%) [5]. In addition to measuring failure rates, patients who underwent revision hip arthroscopy and labral reconstruction reported a more dramatic improvement in postoperative ratings on the Lower Extremity Functional Scale (LEFS) and mHHS, as well as improved pain as reported on the Visual Analogue Scale (VAS) [5]. For example, in patients who underwent revision arthroscopy with allograft labral reconstruction, the mean mHHS improved by 33 points, whereas the mean improvement in the revision repair group was 28 points [5]. In our revision-versus-repair study, we also sub-analyzed complete, circumferential labral grafts to shorter, segmental grafts and found a significantly lower failure rate with the longer grafts [5].

### Direct Comparison

In regard to labral reconstruction and evaluation of clinical outcomes, it is of salient importance to mention that the lead author performed over 3,000 arthroscopic allograft labral reconstructions between July 2009 and February 2020 — both as primary and revision procedures [15]. In 2012, after performing both labral repairs and reconstructions for the two previous years, a concerning rate of failure was noted among patients who had undergone labral repair [15]. As a result, the lead author began exclusively performing labral reconstruction in all cases — a variable which represents a unique feature of his highly specialized practice and has subsequently allowed for investigation and comparison of outcomes in labral repair versus reconstruction on the same patient, where one hip underwent primary repair and the contralateral hip underwent primary reconstruction [26].

In 2018, we published a self-controlled cohort, or case-crossover, study investigating the differences in outcomes between primary labral repair and primary labral reconstruction in the same patient, performed on contralateral hips. We believe this study has provided some of the strongest evidence supporting primary arthroscopic labral reconstruction of the hip [26]. In our 2018 bilateral hip study, we evaluated a unique cohort of patients who had a labral repair on one hip and a labral reconstruction on the other. They were followed for over 2 years (M = 56 months), included 29 patients (58 hips), 23 females and six males, and were an average age of 32.6 years of age (range: 14.9 to 51.6 years) [26]. Their hips were radiographically similar and the only variable in the study was the labral treatment. At a minimum of 2-year follow-up and with 100% patient participation, none of the hips having undergone primary labral reconstruction had failed, whereas 9 labral repairs failed (31%, *p*<.01) [26]. The patients whose labral repairs failed then elected to have a third surgery to convert their failed repair to a reconstruction.

In addition to differences in treatment failure rates, we found that patients who had undergone labral reconstruction noted superior outcomes compared to repair on a number of patient-reported outcomes, such as the mHHS, LEFS, VAS, and with average pain with activities of daily living (ADLs) [26]. Additionally, patients who underwent labral reconstruction in our bilateral hip study demonstrated more notable improvement compared to what has been described as average patient-reported improvements in other studies evaluating the outcomes of labral reconstructions [5,6,26] as well as what has been described as average patient-reported improvements in other studies evaluating the outcomes of labral repair [48]. For example, we found that in patients who underwent primary labral reconstruction, there was an average 33-point improvement on the mHHS [26], as compared to an average 25-point improvement in other relevant studies [20,24,25,28–32,35,36].

### Graft Choice

In a 2016 study [6], we evaluated the outcomes of a front-to-back, circumferential allograft fixation technique for arthroscopic labral reconstruction. This was the first study to validate the use of allograft in labral reconstruction of the hip. In our “front-to-back” study, we found that of the 131 hips, which were followed for a minimum of 2 years, only 18 failed treatment and converted to THA or required revision arthroscopy. Of the remaining 113 hips, all demonstrated improvement in patient-reported outcomes, including an average 34-point increase in postoperative mHHS (*p* < .0001) and an average 27-point increase in postoperative LEFS (*p* < .0001).

### The Issue of Age

In the area of hip arthroscopy, literature as it pertains to outcomes and age has focused on labral repair, not reconstruction. Two recent systematic reviews focused on hip arthroscopy outcomes in patients ages 40 years and older found that while there was an improvement in PROs the conversion to THA was as high as 30%^49,50^. Likewise, another recent study found that hips with a Tönnis grade of more than 1 had as much as a 133% increased risk of converting to THA [51]. The role of age, as well as increased Tönnis grade, has called into question the appropriateness of arthroscopic hip surgery in an older population [4,49–53]. While we support the contraindication of arthroscopic hip surgery in patients with advanced hip arthritis, as it is not amenable to hip preservation, we believe age may be less of a determinant than is generally argued.

As such, in 2020 we published a study comparing outcomes of patients over the age of 40 who underwent primary labral reconstruction or primary labral repair. Our hypothesis contended that aged and chronically diseased labral tissue is compromised and does not heal well with labral repair. Improved results have been demonstrated when this labral tissue is removed, and the patient undergoes a circumferential labral reconstruction. In our “over-40” study, we followed 312 hips for approximately 4 years [4]. Cohorts were divided by age and procedure, including labral reconstruction in patients 40 years and older (*n* = 158), labral repair in patients 40 years and older (*n* = 93), and a control group of labral reconstruction in patients ages 30 to 39 years (*n*=112) [4]. We found that failure was 3.29 times more likely in the over-40 repair group when compared to the over-40 reconstruction group (relative rate, 3.29; *p* = .02), and that there was no difference in failure rates between the 30 to 39 reconstruction group when compared to the over-40 reconstruction group (relative rate, .58; *p* = .37) [4]. Labral repairs in the over-40 groups were found to have failed 22% of the time, whereas primary reconstructions failed only 8% of the time [4]. Likewise, patients in the over-40 reconstruction group demonstrated similar improvements on patient-reported outcomes, including the mHHS, LEFS, and VAS when compared to the 30 to 39 reconstruction group, both of which were superior to the over-40 repair group [4] (*p* < .01). For example, patients in the over-40 reconstruction group reported a 37-point improvement on the mHHS, whereas the over-40 repair group reported a 28-point improvement.

## Conclusion

Considering the positive evidence supporting labral reconstruction, the lead author performs only this procedure and is an advocate for labral reconstruction as both a primary and revision procedure as a more complete solution in the treatment of labral tears and FAI in the presence of irreparable labral tissue. It should also be regarded as the standard for revision hip arthroscopy. However, this stance is considered by some to be controversial as they still relegate labral reconstruction to a salvage operation. The decision to reconstruct the labrum should be made based out of respect for the operation and surgeon experience. When performed poorly, labral reconstruction has the potential to be catastrophic, especially in instances where the acetabular rim is over-resected, thereby resulting in iatrogenic dysplasia. Conversely, when performed well, current evidence has demonstrated a high likelihood of success with labral reconstruction as a primary operation. In direct comparison studies to labral repair, the outcomes with labral reconstruction are similar or better depending on the institution.

The field of hip arthroscopy has grown exponentially in the last decade, and as a specialty, we have seen substantial advancement in the technical abilities of surgeons, and the evolution of the operation itself. Labral reconstruction plays a vital role in hip preservation by re-establishing normal anatomy and function especially in situations where the native labrum cannot be preserved. When compared to labral repair, research has shown that labral reconstruction has the potential to provide more significant improvements in pain and restoration of function. Labral reconstruction should have a role in the practice of every high-volume hip arthroscopist. Current evidence supports our recommendation that arthroscopic circumferential allograft labral reconstruction should be performed in all revision settings and in any instance when surgeons believe the labrum is irreparable. As evidenced by several recently published studies, patients across a broad spectrum of indications who undergo labral reconstruction, whether as a primary procedure or in the revision setting, demonstrate improved outcomes and low rates of failure. While a number of surgical techniques, including a variety of allograft options, have been described in the literature, our research and review of literature supports our recommendation for a circumferential, front-to-back fixation using an iliotibial band allograft, where the graft is measured and cut inside the joint [6, 15]. The lead author welcomes all surgeons interested in learning this technique to come visit.
